# Escape from recognition of SARS-CoV-2 Beta variant spike epitopes but overall preservation of T cell immunity

**DOI:** 10.1126/scitranslmed.abj6824

**Published:** 2022-02-09

**Authors:** Catherine Riou, Roanne Keeton, Thandeka Moyo-Gwete, Tandile Hermanus, Prudence Kgagudi, Richard Baguma, Ziyaad Valley-Omar, Mikhail Smith, Houriiyah Tegally, Deelan Doolabh, Arash Iranzadeh, Lynn Tyers, Hygon Mutavhatsindi, Marius B. Tincho, Ntombi Benede, Gert Marais, Lionel R. Chinhoyi, Mathilda Mennen, Sango Skelem, Elsa du Bruyn, Cari Stek, SA-CIN, Tulio de Oliveira, Carolyn Williamson, Penny L. Moore, Robert J. Wilkinson, Ntobeko A. B. Ntusi, Wendy A. Burgers

**Affiliations:** 1 Wellcome Centre for Infectious Diseases Research in Africa, University of Cape Town; Observatory 7925, South Africa; 2 Institute of Infectious Disease and Molecular Medicine, University of Cape Town; Observatory 7925, South Africa; 3 Division of Medical Virology, Department of Pathology; University of Cape Town; Observatory 7925, South Africa; 4 National Institute for Communicable Diseases of the National Health Laboratory Services; Johannesburg, South Africa; 5 MRC Antibody Immunity Research Unit, School of Pathology, Faculty of Health Sciences, University of the Witwatersrand; Johannesburg, South Africa; 6 KwaZulu-Natal Research Innovation and Sequencing Platform; Durban, South Africa; 7 Groote Schuur Hospital Medical Virology Laboratory of the National Health Laboratory Service; Observatory 7925, South Africa; 8 Department of Medicine, University of Cape Town and Groote Schuur Hospital; Observatory 7925, South Africa; 9 Hatter Institute for Cardiovascular Research in Africa, Faculty of Health Sciences, University of Cape Town; Observatory 7925, South Africa; 10 Department of Infectious Diseases, Imperial College London; W12 ONN, UK; 11 The Francis Crick Institute; London, NW1 1AT, UK

## Abstract

SARS-CoV-2 variants have emerged that escape neutralization and potentially impact vaccine efficacy. T cell responses play a role in protection from reinfection and severe disease, but the potential for spike mutations to affect T cell immunity is incompletely understood. We assessed neutralizing antibody and T cell responses in 44 South African COVID-19 patients infected either with the Beta variant (dominant from November 2020 to May 2021) or infected prior to its emergence (first wave, Wuhan strain), to provide an overall measure of immune evasion. We show that robust spike-specific CD4 and CD8 T cell responses were detectable in Beta-infected patients, similar to first wave patients. Using peptides spanning the Beta-mutated regions, we identified CD4 T cell responses targeting the wild type peptides in 12/22 first wave patients, all of whom failed to recognize corresponding Beta-mutated peptides. However, responses to mutated regions formed only a small proportion (15.7%) of the overall CD4 response, and few patients (3/44) mounted CD8 responses that targeted the mutated regions. Among the spike epitopes tested, we identified three epitopes containing the D215, L18, or D80 residues that were specifically recognized by CD4 T cells, and their mutated versions were associated with a loss of response. This study shows that in spite of loss of recognition of immunogenic CD4 epitopes, CD4 and CD8 T cell responses to Beta are preserved overall. These observations may explain why several vaccines have retained the ability to protect against severe COVID-19 even with substantial loss of neutralizing antibody activity against Beta.

## INTRODUCTION

High levels of ongoing SARS-CoV-2 transmission have led to the emergence of successive new viral variants, which now dominate the pandemic. Variants of concern have been characterized as having increased transmissibility, potentially greater pathogenicity, and the ability to evade host immunity (1). Five such variants of concern have circulated around the world, namely Alpha, Beta, Gamma, Delta, the latter widely replacing many other variants, and more recently Omicron (2–7). A primary concern is whether the immune response generated against ancestral SARS-CoV-2 strains, upon which all approved first generation vaccines are based, still confers protection against variants. The potential threat of reduced vaccine efficacy has prompted swift action from vaccine manufacturers, and adapted vaccines based on other variants have been developed and tested in preclinical and clinical trials (8, 9).

Before the recent emergence of the SARS-CoV-2 Delta variant, the Beta variant, which was first described in South Africa in October 2020 (5), was responsible for >95% of infections in the country and has spread across much of southern Africa (6). It was a concerning variant from an immunological perspective, demonstrating the greatest reduction in neutralization sensitivity to COVID-19 convalescent and vaccinee plasma (10–15), as well as reduced vaccine efficacy (16–18). However, some vaccines have still demonstrated high efficacy against severe COVID-19 after Beta infection (19), suggesting that T cell immunity plays an important role in immune protection, and may mitigate the effect of reduced neutralizing antibody activity.

To date, efforts to characterize immune evasion by SARS-CoV-2 variants have focused mainly on their ability to escape neutralization (10–15). There is limited data addressing whether SARS-CoV-2 variants can evade T cell immunity (20–24) in natural infection or after vaccination. Furthermore, spike-specific T cell responses in COVID-19 patients infected with variant lineages have not been investigated. Here, we determined whether Beta spike mutations affect the recognition of T cell epitopes in patients infected with the ancestral or Beta SARS-CoV-2 lineages. We demonstrate that loss of CD4 T cell recognition does indeed occur in Beta-mutated spike regions, although the majority of the T cell response is maintained. Furthermore, Beta-infected patients mounted comparable spike responses as those infected with earlier strains. These results have important implications for reinfection and vaccine efficacy.

## RESULTS

### T cell responses in patients infected with ancestral strains or Beta

**Fig. 1.**
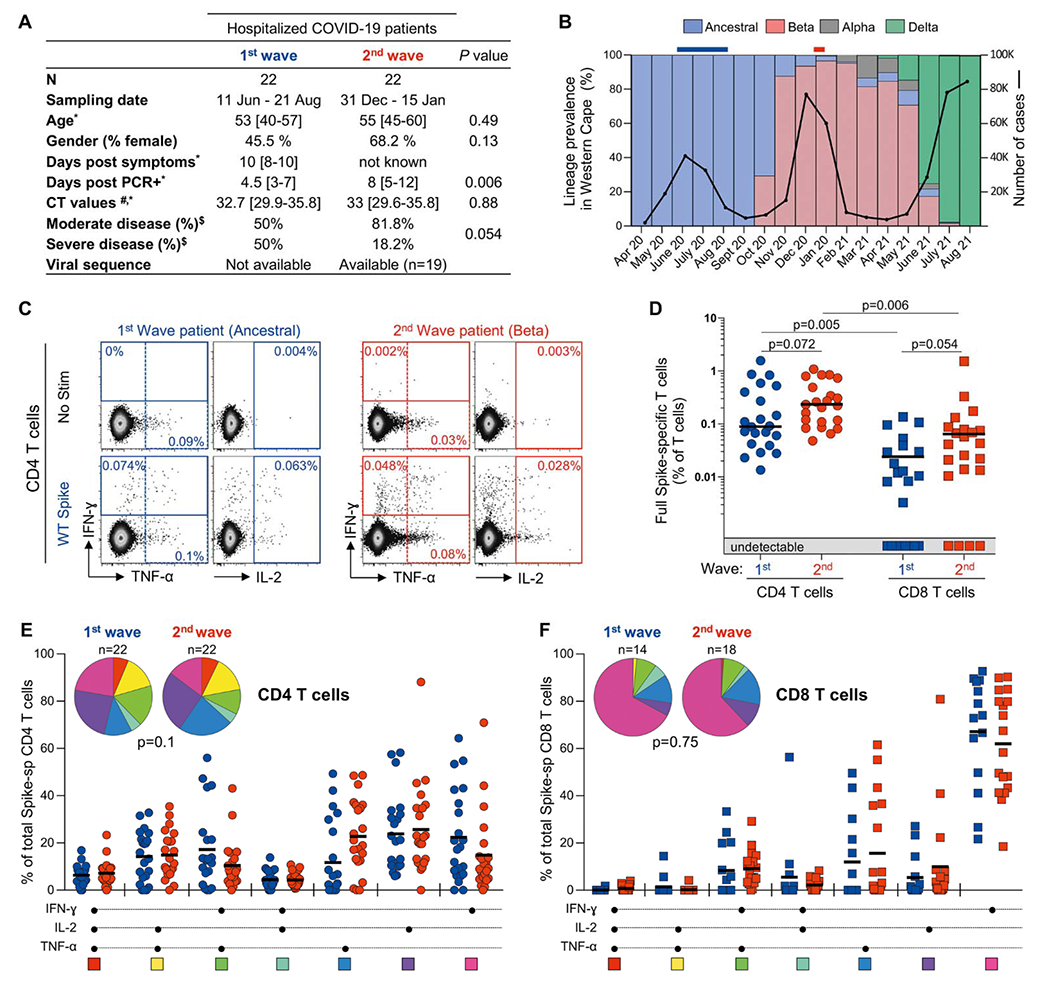
T cell recognition of SARS-CoV-2 spike in first and second wave COVID-19 patients. **(A)** Clinical characteristics of acute COVID-19 patients recruited during the first and second wave of the COVID-19 pandemic in South Africa. *: median and interquartile range.^$^: Disease severity was defined based on oxygen therapy requirement according to the WHO ordinal scale scoring system Moderate (no O_2_ or O_2_ via nasal prongs) or severe (O_2_ via high flow to ECMO).^#^: SARS-CoV-2 polymerase chain reaction (PCR) was performed using the Allplex 2019-nCoV Assay (Seegene). The cycle threshold (CT) value for the N-gene is reported. **(B)** SARS-CoV-2 epidemiological dynamics in the Western Cape (South Africa). Prevalence of SARS-CoV-2 strains is on the left y-axis (based on sequencing 4549 samples). Ancestral strains are depicted in blue, Beta in red, Alpha in grey and Delta in green. Monthly COVID-19 cases are on the right y-axis. Bars above the graph indicate when samples were collected. **(C)** Representative flow cytometry plots of IFN-γ, TNF-α and IL-2 production by CD4 T cells in response to ancestral full spike peptide pool (Full Spike) in one first wave (blue) and one second wave (red) COVID-19 patient. Frequencies of cytokine-producing cells are indicated. **(D)** Frequency of SARS-CoV-2-specific CD4 or CD8 T cells producing IFN-γ, TNF-α or IL-2, in first wave (n = 22, blue) and second wave (n = 22, red) COVID-19 patients. Bars represent medians of responders. Statistical analyses were performed using the Mann-Whitney test between T cell responders from the first and second wave and the Wilcoxon test between CD4 and CD8 responders. **(E)** Comparison of polyfunctional profiles of SARS-CoV-2-specific CD4 T cells in first and second wave patients. **(F)** Comparison of polyfunctional profiles of SARS-CoV-2-specific CD8 T cells in first and second wave patients. The medians are shown. Each response pattern is color-coded, and data summarized in the pie charts. Statistical differences between pies were defined using a permutation test.

SARS-CoV-2 spike-specific neutralizing antibody and T cell responses were measured in hospitalized COVID-19 patients enrolled at Groote Schuur Hospital (Western Cape, South Africa) during the first wave of the COVID-19 pandemic (n = 22), prior to the emergence of the Beta variant, and during the second wave of the pandemic (n = 22), after the Beta variant became the dominant lineage (Fig. 1A). During the first wave, all sequenced virus corresponded to ancestral SARS-CoV-2 lineages (Wuhan and D614G). Conversely, during the second wave, the Beta lineage accounted for >95% of reported SARS-CoV-2 infections at the time of sample collection (Fig. 1B). Beta is defined by nine amino acid changes in the spike protein, and all second wave participants that we sequenced (19/22) had confirmed infection with Beta and harbored 7 to 8 changes associated with the Beta lineage (5) (Fig. S1). Although SARS-CoV-2 viral sequences were not available for patients recruited in June to August 2020 during the first wave, we assumed that all participants were infected with a virus closely related to the ancestral virus, since Beta was first detected in October 2020 in the Western Cape.

First, we compared the magnitude of CD4 and CD8 T cell responses directed at the spike protein of SARS-CoV-2 in first and second wave patients. Using flow cytometry, we measured the production of IFN-γ, TNF-α or IL-2 in response to a peptide pool covering the full ancestral spike protein (‘Full spike’) (Fig. 1C). All participants tested exhibited a CD4 response, with a comparable frequency of spike specific-CD4 T cells in first and second wave patients (*P* = 0.072, Fig. 1D). A detectable spike CD8 response was observed in 63.6% (14/22) of first wave patients and 81.8% (18/22) of second wave patients (*P* = 0.31, Fisher’s exact test). Amongst CD8 responders, the frequency of SARS-CoV-2 spike-specific CD8 T cell response was not significantly different between first and second wave patients (*P* = 0.054). As previously reported (25), the magnitude of the SARS-CoV-2 spike-specific CD8 T cell response was significantly lower compared to the CD4 response in both first and second wave patients (*P* = 0.0005 and *P* = 0.007, respectively). Moreover, there were no significant differences in the frequency of SARS-CoV-2 spike-specific CD4 or CD8 T cells between patients with moderate or severe disease (*P* = 0.3 and *P* = 0.36, respectively). Also, no associations were found between the frequency of spike-specific CD4 or CD8 T cells and days post-PCR positivity (*P* = 0.20, r= 0.28 and *P* = 0.1, r = 0.24, respectively) or days since symptom onset in patients recruited during the first wave (*P* = 0.22, r = 0.28 and *P* = 0.77, r = 0.07, respectively). Lastly, the polyfunctional profiles of SARS-CoV-2 spike-specific CD4 or CD8 T cells were similar between first and second wave patients, with approximately one third of CD4 cells expressing at least two cytokines, while CD8 response was mostly IFN-γ monofunctional (Fig. 1E and F).

**Fig. 2.**
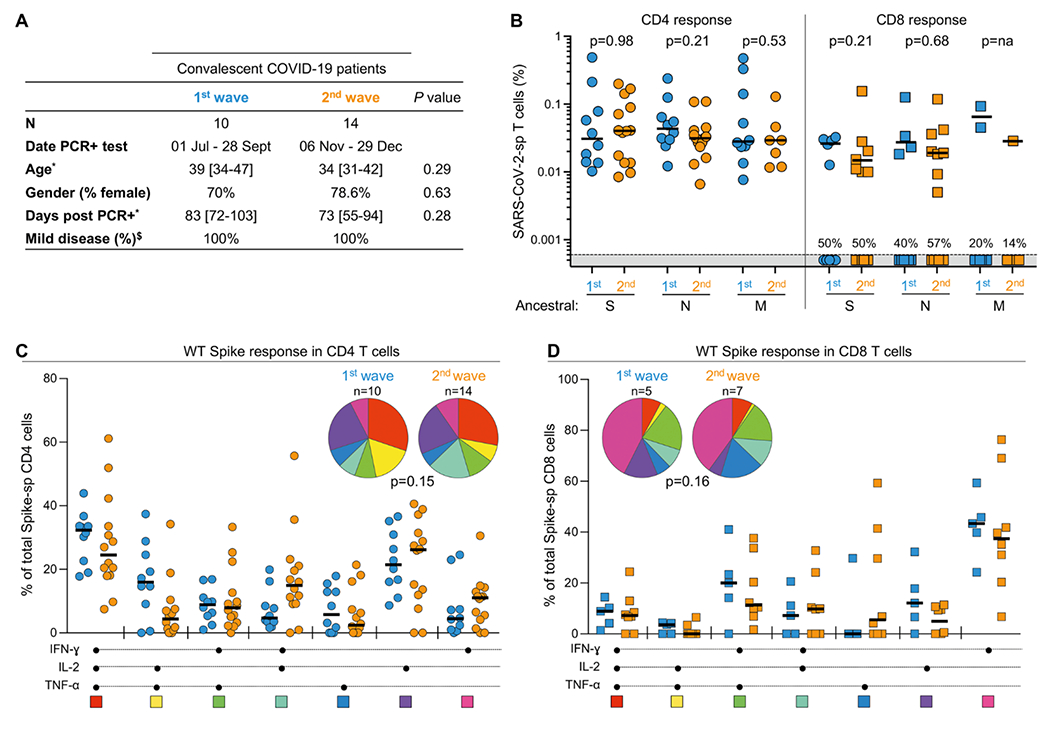
T cell recognition of WT SARS-CoV-2 spike (S). nucleocapsid (N) and membrane (M) proteins in first and second wave convalescent COVID-19 patients. **(A)** Clinical characteristics of convalescent COVID-19 patients recruited during the first and second wave of the COVID-19 pandemic. *: median and interquartile range. ^$^: Disease severity was defined based on oxygen therapy requirement according to the WHO ordinal scale scoring system. **(B)** Summary graph of the frequency of ancestral SARS-CoV-2 S-, N- or M-specific CD4 or CD8 T cells, producing IFN-γ, TNF-α or IL-2, in first wave (n = 10, light blue) and second wave (n = 14, orange) convalescent COVID-19 patients. Due to limited cell availability, T cell responses to M were tested in 10 first wave participants and 7 s wave participants. The proportion of participants exhibiting a detectable CD8 response is indicated. Bars represent medians of responders. Statistical analyses were performed using the Mann-Whitney test. **(C&D)** Polyfunctional profiles of ancestral Spike-specific CD4 and CD8 T cells in first and second wave convalescent COVID-19 patients. Medians and IQR are shown. Each response pattern is color-coded, and data are summarized in pie charts. Statistical differences between pies were defined using a permutation test.

To ascertain whether similar patterns were maintained in convalescent COVID-19 donors, we compared the frequency of ancestral SARS-CoV-2 spike-, nucleocapsid (N)- and membrane (M)-specific CD4 and CD8 T cells in convalescent COVID-19 patients infected during the first wave with the ancestral strain (n = 10) or during the second wave, when Beta dominated (n = 14) (Fig. 2A). As for acute COVID-19 patients, the magnitude and polyfunctional profiles of ancestral SARS-CoV-2 spike-specific CD4 and CD8 T cell responses were comparable between convalescent individuals infected during the first or the second wave (Fig. 2B, C and D). Similar results were observed for CD4 responses against the SARS-CoV-2 N and M proteins (Fig. 2B). We also compared the profiles of spike-specific T cell responses cross-sectionally between acute and convalescent COVID-19 patients. Our data show that the frequency of spike-specific CD4 T cells is significantly lower in convalescent compared to acutely infected patients, regardless of the infecting strains (*P* = 0.03 for WT and *P* < 0.0001 for Beta) (Fig. S2A), which is likely related to the contraction of antigen-specific responses following viral clearance (26). For spike-specific CD8 T cell responses, a lower frequency was observed only between acute and convalescent patients from the second wave (*P* = 0.034). Lastly, an increase in the polyfunctional profile of both spike-specific CD4 and CD8 T cells was observed in convalescent COVID-19 patients compared to those in the acute phase of infection, characterized by a substantial increase in IFN-γ, TNF-α and IL-2 co-expressing cells for spike-specific CD4 T cells and a significant reduction of IFN-γ monofunctional cells for spike-specific CD8 T cells (Fig. S2B and C). Overall, these data are in accordance with a recent report showing that T cell responses directed at the SARS-CoV-2 spike protein in convalescent COVID-19 donors infected with SARS-CoV-2 ancestral strain were not substantially affected by mutations found in SARS-CoV-2 variants (24), and we further show that there is no overall dampening of T cell responses or change in functional profiles to the three immunogenic structural proteins of SARS-CoV-2 in those infected with Beta.

### CD4 T cell targeting of variant spike epitopes

**Fig. 3.**
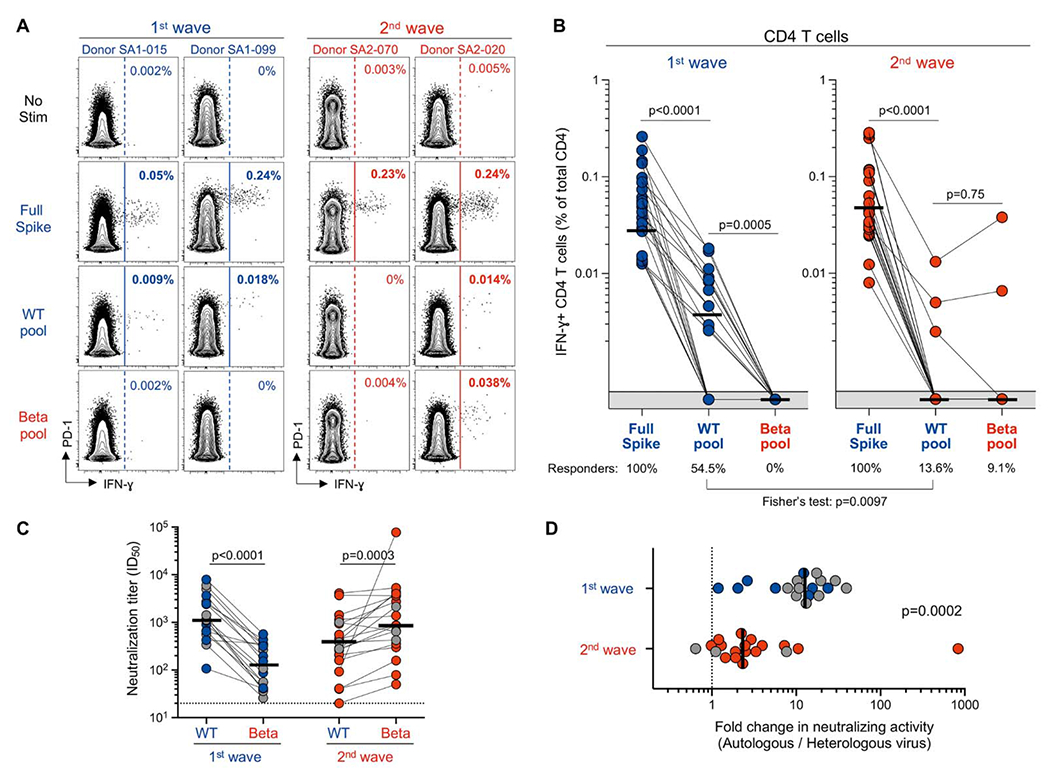
Loss of recognition of SARS-CoV-2 Beta variant epitopes and neutralizing antibody responses. **(A)** Representative flow cytometry plots of IFN-γ production by CD4 T cells in response to ancestral full spike peptide pool (Full spike), and smaller pools spanning the mutated regions of ancestral (WT pool) or Beta spike (Beta pool) in two first wave (blue) and two second wave (red) COVID-19 patients. Frequencies (%) of IFN-γ positive cells are indicated. **(B)** The frequency of IFN-γ-producing SARS-CoV-2-specific CD4 T cells in first wave (n = 22, left) and second wave (n = 22, right) COVID-19 patients. The proportion of patients exhibiting a detectable response to the different peptide pools (i.e., responders) is indicated at the bottom of each graph. **(C)** Plasma samples from COVID-19 patients recruited during the first (n = 18) or the second wave (n = 19) were tested for neutralization cross-reactivity against ancestral or Beta pseudoviruses. The threshold of detection was a 50% inhibitory dilution (ID_50_) of 20. Gray dots indicate patients who displayed a detectable CD4 T cell response to WT pool, selectively covering the variable regions of spike, and lost recognition to the Beta pool. Neutralization data on the second wave cohort are from (25). **(D)** Fold-change in neutralization titers is shown for data in **c**. Bars represent medians. Statistical analyses were performed using the Wilcoxon test and the Fisher’s exact-squared test.

Since Beta-associated mutations occur only at a few residues of the spike protein, we assessed the recognition of peptide pools selectively spanning the variable regions of spike, one composed of the ancestral peptides (‘WT pool’) and the other Beta-mutated peptides (‘Beta pool’) (Table S1). Sample availability enabled us to perform these experiments in the acute COVID-19 cohort. Due to elevated TNF-α background observed in unstimulated cells (Fig. 1C), we focused on IFN-γ producing cells to measure T cell response to the smaller peptide pools. We previously described that acute COVID-19 patients had SARS-CoV-2-specific CD4 T cells characterized by elevated expression of PD-1 (27). Thus, PD-1 was included in our flow cytometry panel to ensure that the phenotypic profile of CD4 T cells responding to the variable spike epitopes were consistent with our previous findings. In patients recruited during the first wave, IFN-γ CD4 T cell responses to the WT pool were detectable in 54.5% (12/22) patients (Fig. 3A and B). In those who mounted responses, the magnitude of the WT pool response was ~ 6.4-fold lower than full spike responses (median: 0.0075% vs 0.048%, respectively, *P* < 0.0001). In the 12 participants responding to the WT pool, the overall median relative contribution of WT epitopes located at spike mutation sites to the total spike-specific CD4 T cell response was 15.7%, ranging from 5.7% to 24%. This suggests that the majority of SARS-CoV-2 spike-specific CD4 T cell responses are directed against conserved epitopes between the ancestral and Beta lineage. When we tested the corresponding Beta pool, all 12 of the first wave WT pool responders failed to cross-react with the mutated peptides from Beta (Fig. 3B, left panel). These results show that Beta-mutated epitopes were no longer recognized by CD4 T cells targeting WT epitopes, demonstrating that this loss of recognition is likely mediated by variant mutations. This is broadly consistent with recent data from mRNA vaccinees, where full spike pools containing Beta mutated peptides detected T cell responses that were diminished by 30% compared to ancestral spike, revealing that the mutated sequences mediate differential recognition but make up a minor contribution to the overall spike-specific T cell response (24).

We next measured peptide responses in patients infected with the Beta lineage. The Beta pool was not readily recognized by patients infected with the homologous variant (2/22; 9.1%) (Fig. 3B, right panel). A single donor had a detectable response to the WT but not Beta pool. These data suggest that mutations in Beta spike epitopes likely alter epitope binding to restricting HLA molecules, consistent with the loss of recognition of Beta-mutated peptides by T cells in first wave patients.

In order to obtain an overall measure of immune escape in our participants, we measured their neutralizing antibody responses to the ancestral and Beta spike proteins (Fig. 3C and D). As we showed previously (13), in patients infected with the ancestral strains (first wave), a considerable loss of neutralization activity was observed against Beta (median fold change: 12.7, IQR: 7.3-18.8). In contrast, patients infected with Beta (second wave) retained a substantial capacity to neutralize ancestral virus, as shown by a moderate reduction in neutralizing activity (median: 2.3, IQR: 1.3-3.9). Of note, in the six first wave patients where loss of cross-neutralization was profound (titer <100), it is reassuring that the T cell response was relatively intact. We found no association between the frequency of SARS-CoV-2 spike-specific CD4 T cell responses and neutralizing activity (Fig. S3A), consistent with an earlier study (28). Moreover, comparable frequencies of SARS-CoV-2-specific CD4 T cells were observed, irrespective of the extent of the loss of neutralizing activity against heterologous virus (Fig. S3B).

### CD8 T cell targeting of variant spike epitopes

**Fig. 4.**
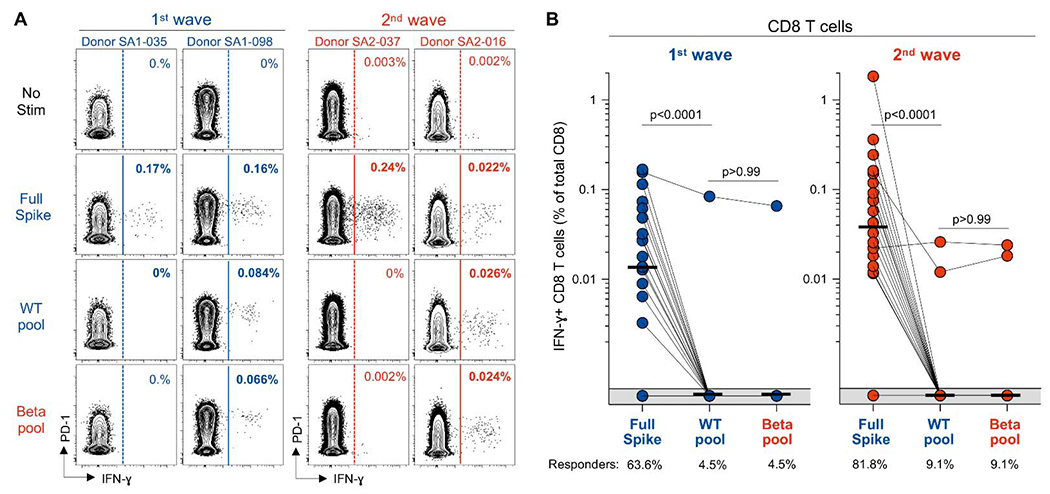
Infrequent recognition of SARS-CoV-2 ancestral or Beta variant spike epitopes by CD8 T cells. **(A)** Representative flow cytometry plots of IFN-γ production by CD8 T cells in response to ancestral full spike peptide pool (Full Spike), and pools covering the mutated regions of ancestral spike (WT pool) or Beta spike (Beta pool) in two first wave (blue) and two second wave (red) COVID-19 patients. Frequencies (%) of IFN-γ positive cells are indicated. **(B)** Frequency of IFN-γ-producing SARS-CoV-2-specific CD8 T cells in first wave (n = 22, left) and second wave (n = 22, right) patients. The proportion of responders is indicated. Bars represent medians. Statistical analyses were performed using the Wilcoxon test.

We next defined the recognition of WT and Beta peptide pools by CD8 T cells (Fig. 4A). Regardless of the infecting SARS-CoV-2 lineage, peptides covering the spike mutation sites were rarely recognized by CD8 T cells, with only 3/44 (6.8%) patients exhibiting a CD8 response, one in the first wave cohort and two in the second wave cohort. Thus, in contrast to CD4 T cells, the regions in which Beta mutations occur are not commonly targeted by CD8 T cells. Moreover, in these three patients, the frequency of IFN-γ-producing CD8 T cells was comparable between WT and Beta pool stimulation, indicating that mutations did not affect epitope recognition (Fig. 4B). Overall, these data indicate that Beta mutations do not affect CD8 T cell responses in our cohort.

### Mapping of spike variable epitopes targeted by T cells

**Fig. 5.**
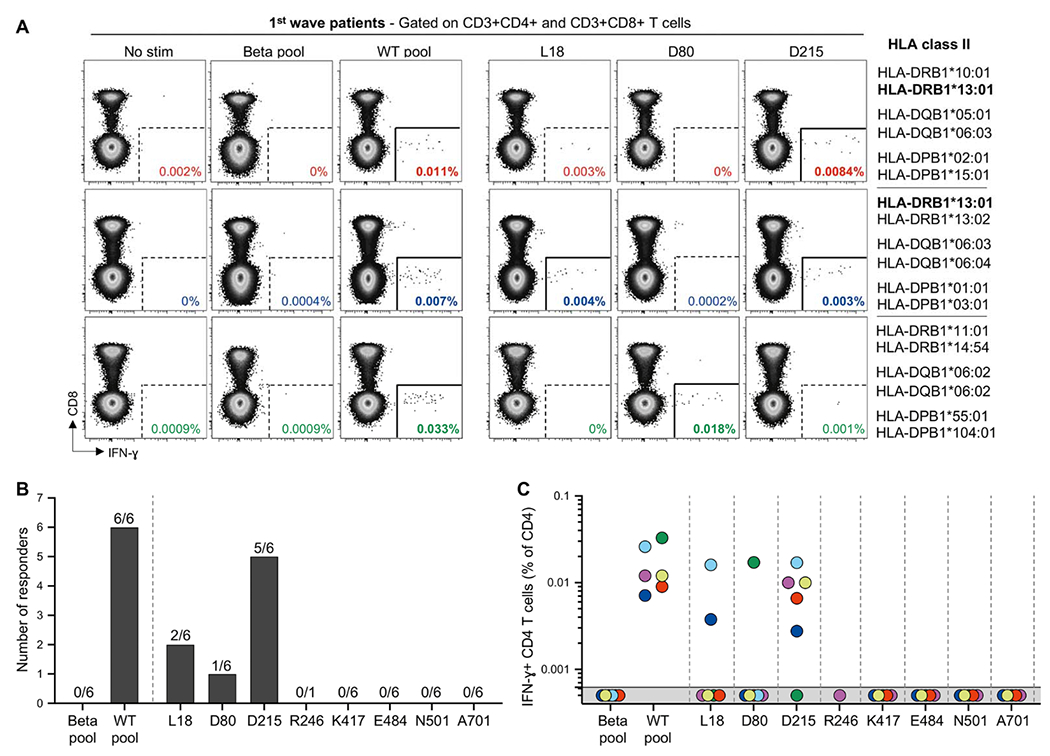
Identification of SARS-CoV-2 spike epitopes targeted by CD4 T cells. **(A)** Representative flow plots of IFN-γ production by CD8 and CD4 T cells in response to the Beta pool, WT pool and peptide pairs containing the spike 6-25 sequence (containing L18), the 73-92 sequence (containing D80) and the 206-225 sequence (containing D215) in three first wave patients. HLA Class-II alleles of each participant are listed on the right. **(B)** Number of tested first wave participants (n = 6) exhibiting a response to Beta pool, WT pool and each of the peptide pairs tested individually. **(C)** Frequency of IFN-γ+ CD4 T cells in response to indicated stimuli. Each participant is depicted by a different color.

To gain deeper insight into the recognition of variable spike epitopes by CD4 cells in patients responding to the WT pool, responses to individual epitopes were measured in first wave COVID-19 patients (Fig. 5A). Amongst the six tested patients, a response to the spike 206-225 region (containing D215) was observed in 5 out of 6 patients, two of which also displayed a response to spike 6-25 region (containing L18). Moreover, a response toward the spike 73-92 region (containing D80) was detected in one participant (Fig. 5B). Mutation of these regions (L18F, D80A and D215G) resulted in a loss of CD4 response (Fig. 3B and Fig. 5A). No CD4 T cell responses to epitopes containing the K417, E484, N501 or A701 residues were observed (Fig. 5B and C).

To identify the potential HLA restriction associated with the recognition of the L18, D80 and D215 epitopes, predicted HLA class II restriction for each epitope was defined *in silico* (Table S2) and compared to HLA class II molecules expressed in our study cohort (Table S3). We identified that the D215 epitope was restricted by DRB1*03:01, DRB1*03:02 or DRB1*13:01; and the D215G mutation is predicted to be associated with a loss of response in those three alleles (Data file S1), as previously reported (29). Nine first wave patients carried one of these alleles, eight of whom exhibited a response to the D215 epitopes (n = 5) or the WT pool (n = 3). No matching alleles were predicted for the L18 and D80 epitopes, despite CD4 responses to the spike regions 6-20, 11-25 and 78-92 having been previously reported previously (30, 31).

Due to limited availability of samples, we could not test all WT pool responders for single epitope responses. However, based on predicted HLA class II restriction, we hypothesized that the peptide ^236^TRFQTLLALHRSYLT^250^ (WT version of the 242-244del/R246I) may be an immunogenetic epitope, as previously described (30, 32, 33) restricted by DRB1*15:01, DRB1*15:03, DRB1*01:01 or DRB1*14:25 (Table S4). The 242-244del/R246I mutation is predicted to be associated with a loss of response to three of those alleles (DRB1*15:01, DRB1*15:03, and DRB1*14:25), while DRB1*01:01 retains its ability to bind the Beta-mutated epitope (^236^TRFQTLHISYLTPGD^250^, 242-244del/R246I) based on the predicted IC50 and percentile rank value (Data file S1). Of note, 4 out of 6 alleles of interest (DRB1*03:01, DRB1*03:02, DRB1*13:01, DRB1*15:01, DRB1*15:03) exhibited comparable frequency distributions in first and second wave patients, while DRB1*01:01 and DRB1*14:25 were identified in only two patients from wave 1 (Data file S2). Of note, in first wave patients who did not mount a response to the WT pool (n = 10 out of 22, Fig. 4B), only two expressed an HLA-DRB1 allele associated with the recognition of the D215 or R246 epitopes. The absence of response in these two donors could be due to the limited sensitivity of the flow cytometry assay used to measure T cell responses in this study.

In patients infected with Beta, three individuals exhibited a response to the WT pool, two of whom also responded to the Beta pool. Based solely on *in silico* predicted HLA class II restriction analysis, it was not possible to infer potential targeted peptides, as no specific epitopes could be associated with the HLA class II alleles carried by these patients. Of note, the viral sequence of all three did not have the L18A mutation (maintaining a lysine in position 18, characteristic of the WT strain). Additionally, in one of these responders, no D215G substitution was observed, but this specific individual did not carry any of the alleles associated with the recognition of the D215-containing epitope (Table S4).

Regarding specific spike epitopes recognized by CD8 T cells, only three individuals exhibited a CD8 T cell response to the WT pool and comparable responses were obtained with the Beta pool (Fig. 4). All epitopes were tested *in silico* for predictive binding to HLA class I variants (HLA-A and HLA-B) expressed in the cohort (Table S5). The epitope ^84^LPFNDGVYF^92^ showed the highest ranking for HLA-B*53:01, HLA-B*35:05 and HLA-B*35:30. Since CD8 responding participants carried the HLA-B*53:01 (SA2-016) or HLA-B*35:05 (SA1-098 and SA2-084) allele (Data file S3), this strongly suggests that the ^84^LPFNDGVYF^92^ epitope is recognized by those participants. This confirms results reported by Tarke *et al*. (31) and further demonstrates that ^84^LPFNDGVYF^92^ is also restricted by HLA-B*35:05. Moreover, as this predicted 9mer epitope is conserved between the ancestral strain and Beta variant and does not include the beta-mutated residue (D80A), it is unsurprising that the observed CD8 response is comparable when stimulation is performed using the WT or the Beta pool. Finally, these two alleles (B*53:01 and HLA-B*35:05) were found in only four participants (Table S5), three of whom exhibited a CD8 response to the WT and Beta pool.

## DISCUSSION

We demonstrate that infection with the Beta variant results in robust T cell responses, comparable to responses elicited to ancestral strains. We also demonstrate that the recognition of epitopes by CD4 T cells targeting variable spike regions is affected by Beta spike mutations in patients infected with ancestral lineages. However, the loss of recognition of Beta mutated spike epitopes had a minor impact on the overall CD4 Th1 cell response. Moreover, CD8 T cell responses to spike were unaffected by mutations in Beta.

We focused our analysis on spike, because specific mutations or deletions within or outside of T cell epitopes can lead to lack of cross-recognition, or loss of presentation, and may have important implications for vaccine protection. However, recent studies have revealed a more global strategy employed by SARS-CoV-2 variants to potentially evade immunity. The suppression of innate immune responses was demonstrated for the Alpha variant, as well as interferon resistance in vitro (34, 35). Thus, given the possibility that variant mutations may have broader effects on adaptive responses, we also examined T cell responses to other dominant targets, namely the nucleocapsid and membrane proteins (26, 28, 31, 36). We detected similar T cell response frequencies between first and second wave convalescent donors for both CD4 and CD8 T cells, irrespective of the SARS-CoV-2 protein examined, suggesting that there was not a general dampening of T cell responses to Beta. Altogether, we confirm that infection with Beta does not significantly affect the overall recognition or functional profile of T cell responses to the ancestral virus in either acute or chronic infection.

It is of interest to determine whether specific mutations in SARS-CoV-2 variants may lead to evasion of cellular immunity, as has been demonstrated for neutralizing antibodies. Having demonstrated that ancestral versions of peptides mutated in Beta were targeted by CD4 T cells from first wave participants, and there was a loss of recognition of the Beta peptides, we reasoned that one or more epitopes in the Beta pool may be affected by variant mutations. We identified three epitopes containing the D215, L18, or D80 residues that were specifically recognized by CD4 T cells, and mutated versions in Beta were associated with a loss of response. HLA genotyping revealed the predicted MHC class II alleles restricting the D215 epitope, and *in silico* analyses confirmed that mutations would no longer be restricted by those alleles. This provides important information regarding mutations occurring in SARS-CoV-2 variants, the predicted epitopes within which they are located, immune evasion properties associated with them, and their restricting alleles. Of note, the L18F mutation is of importance as it is a frequently observed mutation, with a 4% cumulative prevalence in all SARS-CoV-2 sequences in GISAID (37), present in Gamma and in a number of other lineages, in particular B.1.177 that circulated widely in Europe (38). L18F is expected to have a detrimental impact on antibody binding, thus the mutation could result from selective pressure from both antibodies and T cells. D215, located in the epitope most frequently targeted by first wave patients of the three epitopes we identified, is mutated to G in Beta, which is shared by the C.1.2 variant (39), a highly mutated variant under monitoring, as well as B.1.616 and AT.1 lineages, all of which occur at frequencies <0.5% worldwide (40). These observations further underscore the limited impact these mutations may have on the CD4 T cell response at a global level.

We were unable to confirm class II HLA restriction of the peptides containing the L18 and D80 residues, with the predicted HLA molecules for the L18 epitope not matching those expressed in our cohort, and no restricting alleles predicted for the D80-containing peptide. Epitopes containing these residues have been described in other studies, without presenting HLA restriction (30, 31).This is likely due to the limited accuracy of Class II prediction algorithms. An additional CD4 epitope containing the R246 residue is a likely target for which HLA binding is abrogated in the Beta variant for particular Class II alleles, and may have also contributed to targeting of the WT pool. However, limited cell availability prevented us from experimentally confirming the targeting of this epitope in our cohort, but several studies have confirmed targeting of this region (30, 32, 33).

We found that spike-specific CD8 responses were not affected by mutations in Beta in our cohort. A single epitope (residues 84-94 of spike) was predicted to account for the CD8 response to the WT or Beta pool in three individuals in the cohort. Consistent with the recognition of both WT and mutated pools, the mutation fell outside the core binding motif for the predicted restricting Class I HLA molecules expressed by these donors. Overall, our results emphasize that the HLA repertoire in individuals determined whether they were impacted by mutations in variant epitopes, rather than the mutated epitopes dictating population-wide effects, as observed with certain key neutralizing antibody epitopes in variants of concern. These observations further emphasize that HLA polymorphism will likely limit the impact of T cell escape on SARS-CoV-2 immunity to viral variants. Two possible scenarios could change this: 1) the mutation of dominant epitopes (32) or those broadly restricted (‘promiscuous epitopes’) by multiple commonly-expressed alleles (29), or 2) if accumulation of mutations associated with T cell evasion occurs in variants. To date, neither of these have occurred.

This work extends our recent findings characterizing neutralizing antibody responses elicited by Beta (13, 41). Neutralization resistance for Beta, Gamma and Delta confers the ability to evade antibodies after infection and vaccination, to varying degrees (11, 42–44). Beta is approximately 10-fold more resistant to convalescent plasma and sera from vaccinated individuals than ancestral SARS-CoV-2 (14, 15, 45). Comparative analyses of SARS-CoV-2 variants demonstrated that Beta is the most refractory to neutralization of all the VOC that have emerged to date (12, 46, 47), however early indications are that Omicron will result in greater escape from neutralization (48).

Recent studies examining vaccine-induced neutralizing antibodies (nAb) and vaccine efficacy demonstrate that nAb titers are a correlate of protection (49, 50). The demonstration of an antibody correlate does not preclude a contribution from other immune components for protection. CD4 T cell responses are required for strong Ab responses, and CD8 T cells play an important role in the context of sub-optimal antibody titers in a macaque model (51). Multiple mechanisms, involving nAb, CD4 and CD8 T cells acting in a coordinated manner appear to effectively control established infection (52).

In contrast to neutralizing antibody epitopes, T cell epitopes are abundantly located across the spike protein (30, 31, 53–55). Regions in spike most frequently targeted by CD4 T cells are the N-terminal domain of both the S1 and S2 subunits, with the receptor binding domain (RBD) being relatively epitope-poor (31). This is consistent with the three CD4 epitopes we identified in this study. In contrast, CD8 T cell epitopes are broadly distributed across spike (31, 53). Sustained efforts to map epitopes in spike, particularly in a range of populations encompassing greater HLA diversity will be beneficial for evaluating the effect on T cell immunity for mutations that may arise in future VOC. Thus, it is unsurprising that Beta retains the ability to generate strong T cell immune responses, as Beta spike mutations are limited to a few residues.

Viral evasion of cytotoxic T lymphocyte or T helper recognition may result in delayed clearance of infected cells, or inadequate help provided to B cells, influencing the antibody response. Viral escape from specific SARS-CoV-2 CD8 epitopes has recently been described, in spike, nucleocapsid and ORF3a (20, 21, 23). Both CD4 and CD8 T cells can exert selective pressure on viruses resulting in mutational escape, thereby driving viral evolution. In addition to their role in supporting the maturation of B cells and CD8 T cell responses, CD4 T cells may play additional antiviral roles, including directly lysing infected cells (56). Indeed, transcriptomic profiles of SARS-CoV-2-specific CD4 T cells demonstrated a subset expressing transcripts for cytotoxic molecules (57). Abundant populations of cytotoxic CD4 have been described in the lungs in COVID-19 patients (58), where they may participate in viral clearance. In addition to direct selective pressure, mutations occurring in response to immune pressure from neutralizing antibodies or associated with increased viral infectivity (23) could coincide with T cell epitopes, thus representing ‘collateral damage’ for the T cell response.

Our study had several limitations. Although convenient for mapping approaches, it has been demonstrated that 15mer peptides are not optimal for all HLA class I-restricted T cells (59). Approaches using optimal CD8 epitopes (25, 53) may have yielded greater sensitivity to detect CD8 responses. Furthermore, examining responses to Beta in the context of full mutated spike (22, 24) would corroborate our findings regarding the degree to which the overall spike T cell response is affected by mutations.

In conclusion, although Beta no longer has significant prevalence compared to Delta and the highly mutated Omicron in residues that are key for antibody recognition, these results are relevant in advancing our understanding of the cross-reactive potential of T cell immunity in the context of viral variability and highlight the importance of monitoring both antibody and T cell responses to emerging SARS-CoV-2 variants. We demonstrate a limited effect of viral mutations on T cell immunity which may explain why, despite substantial loss of neutralizing antibody activity against Beta and Delta, vaccines have retained the ability to protect against severe COVID-19. We and others have shown that vaccine-induced T cell immunity effectively recognizes SARS-CoV-2 variants (24, 60–63). While second generation vaccines based on SARS-CoV-2 variants are desirable, they may not be needed to generate improved T cell responses.

## MATERIALS AND METHODS

### Study Design

Hospitalized patients with PCR-confirmed acute COVID-19 were enrolled at Groote Schuur Hospital (Cape Town, Western Cape, South Africa) between June 11^th^ and August 21^st^, 2020 (first wave, n = 22) and between December 31^st^, 2020 and January 15^th^, 2021 (second wave, n = 22). The clinical characteristics of participants are summarized in Fig. 1A Clinical folders were consulted for all second wave patients and none showed evidence of prior symptomatic COVID-19. Blood samples were obtained a median of 4.5 days [interquartile range (IQR): 3-7] after a positive PCR test for SARS-CoV-2 for first wave patients, and 8 days [IQR: 4-16] for second wave patients. Viral sequences were available for 19/22 s wave participants (Fig. S1). T cell responses were assessed by stimulating PBMC with peptide pools spanning full-length spike or smaller pools covering the regions mutated in Beta, followed by intracellular cytokine staining and flow cytometry (Fig. S4). Additionally, convalescent COVID-19 patients infected with the ancestral SARS-CoV-2 strain or Beta were included in this study. Samples were obtained a median of 98 days [IQR: 79-110] after a positive PCR test for first wave participants, and 67 days [IQR: 54-105] for second wave participants (Fig. S2A). The study was approved by the University of Cape Town Human Research Ethics Committee (HREC: 207/2020 and R021/2020) and written informed consent was obtained from all participants.

### SARS-CoV-2 spike whole genome sequencing

Whole genome sequencing of SARS-CoV-2 was performed using nasopharyngeal swabs obtained from 19 of the hospitalized patients recruited during the second wave.

Sequencing was performed as previously published (41). Briefly, cDNA was synthesized from RNA extracted from swabs using the Superscript IV First Strand synthesis system (Life Technologies) and random hexamer primers. Whole genome amplification was performed by multiplex PCR using the ARTIC V3 protocol (https://www.protocols.io/view/ncov-2019-sequencing-protocol-v3-locost-bh42j8ye). PCR products were purified with AMPure XP magnetic beads (Beckman Coulter) and quantified using the Qubit dsDNA High Sensitivity assay on the Qubit 3.0 instrument (Life Technologies). The Illumina® DNA Prep kit was used to prepare indexed paired end libraries of genomic DNA. Sequencing libraries were normalized to 4 nM, pooled, and denatured with 0.2 N sodium hydroxide. Libraries were sequenced on the Illumina MiSeq instrument. The quality control checks on raw sequence data and the genome assembly were performed using Genome Detective 1.132 (https://www.genomedetective.com) and the Coronavirus Typing Tool (64). The initial assembly was polished by aligning mapped reads to the references and filtering out low-quality mutations using bcftools 1.7-2 mpileup method. Mutations were confirmed visually with bam files using Geneious software (Biomatters Ltd). Phylogenetic clade classification of the genomes in this study consisted of analyzing them against a global reference dataset using a custom pipeline based on a local version of NextStrain (https://github.com/nextstrain/ncov) (65). The workflow performs alignment of genomes, phylogenetic tree inference, tree dating and ancestral state construction and annotation. Phylogenetic trees were visualized using ggplot and ggtree (66). GISAID accession numbers are as follows: EPI_ISL_1040644, 1040645, 1040646, 1040650, 1040654, 1040656, 1040659, 1040672, 1040683, 1040692, 1040696, 1040697, 1040698, 1040707, 1040714, 1040716, 1040754, 1040758, 1534362.

### Ancestral (wild type) and Beta variant SARS-CoV-2 peptides

To assess the response to the full length SARS-CoV-2 spike protein, we combined two commercially available peptide pools (PepTivator®, Miltenyi Biotech) including: i) a pool of peptides (15mers with 11 aa overlap) covering the ancestral N-terminal S1 domain of SARS-CoV-2 (GenBank MN908947.3, Protein QHD43416.1) from aa 1 to 692 and ii) a pool of peptides(15mers with 11 aa overlap) covering the immunodominant sequence domains of the ancestral C-terminal S2 domain of SARS-CoV-2 (GenBank MN908947.3, Protein QHD43416.1) including aa 683-707, aa 741-770, aa 785-802, and aa 885-1273. Pools were resuspended in distilled water at 50 μg/mL. Individual peptides (15mers with 11 aa overlap) spanning ancestral or Beta spike mutation sites (L18F, D80A, D215G, del 242-244, R246I, K417N, E484K, N501Y and A701V) were synthesized (GenScript) and individually resuspended in dimethyl sulfoxide (DMSO; Sigma-Aldrich) at 20 μg/mL. Peptide sequences are provided in Table S1, which also indicates where their recognition has been previously described (30, 31, 55). Ancestral or Beta pools (16 peptides) selectively spanning the mutated regions were created by pooling aliquots of these individual peptides at a final concentration of 160 μg/mL. To assess T cell responses to SARS-CoV-2 nucleocapsid and membrane proteins, commercially available peptide pools (15mers with 11 aa overlap, PepTivator®, Miltenyi) covering the complete sequence of the SARS-CoV-2 membrane glycoprotein (M, GenBank MN908947.3, Protein QHD43419.1) or nucleocapsid (N, GenBank MN908947.3, Protein QHD43423.2) were used.

### Isolation of peripheral blood mononuclear cells (PBMC)

Blood was collected in heparin tubes and processed within 3 hours. PBMC were isolated by density gradient sedimentation using Ficoll-Paque (Amersham Biosciences) as per the manufacturer’s instructions and cryopreserved in freezing media consisting of heat-inactivated fetal bovine serum (FBS, Thermo Fisher Scientific) containing 10% DMSO and stored in liquid nitrogen.

### Cell stimulation and flow cytometry staining

Cryopreserved PBMC were thawed, washed and rested in RPMI 1640 containing 10% heat-inactivated FBS for 4 hours. PBMC were seeded in a 96-well V-bottom plate at ~2 × 10^6^ PBMC per well and stimulated with SARS-CoV-2 M or N peptide pools (4 μg/mL), SARS-CoV-2 spike peptide pools: full spike pool (4 μg/mL), and ancestral and Beta pools selectively spanning the mutated regions (4 μg/mL). All stimulations were performed in the presence of Brefeldin A (10 μg/mL, Sigma-Aldrich) and co-stimulatory antibodies against CD28 (clone 28.2) and CD49d (clone L25) (1 μg/mL each; BD Biosciences). As a negative control, PBMC were incubated with co-stimulatory antibodies, Brefeldin A and an equimolar amount of DMSO (0.15%).

After 16 hours of stimulation, cells were washed, stained with LIVE/DEAD Fixable Near-IR Stain (Invitrogen) and subsequently surface stained with the following antibodies: CD4 BV785 (OKT4, Biolegend), CD8 BV510 (RPA-8, Biolegend), PD-1 PE (J105, eBioscience). Cells were then fixed and permeabilized using a Transcription Factor Fixation buffer (eBioscience) and stained with CD3 BV650 (OKT3), IFN-γ BV711 (4S.B3), TNF-α PE-cy7 (MAB11) and IL-2 PE-Dazzle (MQ1-17H12) from Biolegend. Finally, cells were washed and fixed in 1% formaldehyde in PBS. Samples were acquired on a BD LSR-II flow cytometer and analyzed using FlowJo (v9.9.6, FlowJo LLC). The gating strategy is presented in Fig. S5. A cytokine response was defined as positive when the frequency of cytokine produced in stimulated wells was at least twice the background of unstimulated cells. All summary data are presented after background subtraction. For the identification of specific spike epitopes, five acute COVID-19 patients and one convalescent donor were tested.

### SARS-CoV-2 pseudovirus based neutralization assay

SARS-CoV-2 pseudotyped lentiviruses were prepared by co-transfecting the HEK 293T cell line with the SARS-CoV-2 614G spike (D614G) or SARS-CoV-2 Beta spike (L18F, D80A, D215G, K417N, E484K, N501Y, A701V, 242-244 del) plasmids with a firefly luciferase encoding lentivirus backbone plasmid. The parental plasmids were provided by Drs Elise Landais and Devin Sok (IAVI). For the neutralization assays, heat-inactivated plasma samples were incubated with SARS-CoV-2 pseudotyped virus for 1 hour at 37°C, 5% CO_2_. Subsequently, 1x10^4^ HEK293T cells engineered to overexpress ACE-2, provided by Dr Michael Farzan (Scripps Research Institute), were added and the incubated at 37°C, 5% CO_2_ for 72 hours, upon which the luminescence of the luciferase gene was measured. CB6 and CA1 monoclonal antibodies were used as controls.

### HLA typing

Genomic DNA was isolated from PBMC using standard techniques (Qiagen). Amplicons for class I (HLA-A, B and C) and II (DRB1, DQB1 and DPB1) HLA loci were generated using the NGSgo®-MX6-1 multiplex PCR (GenDX), according to the manufacturer’s instructions. Sequencing libraries were prepared using the NGSgo-LibrX kit (GenDX), dual indexed using the NGSgo-IndX kit (GenDX) and pooled, according to the manufacturer’s instructions. Pooled libraries were loaded at 12 pM on a MiSeq Micro flow cell (Illumina) and run using a MiSeq reagent kit V2 (Illumina). Paired-end sequencing was performed on the MiSeq NGS platform (Illumina), 151 cycles in each direction. HLA typing calls were made using the NGS-engine HLA typing software package (Version 2.22, GenDX) along with the 3.44.1 version of the IPD-IMGT/HLA database. HLA class II and class I genotypes are presented in Table S3 and Table S5. The frequency distributions of HLA-DRB1, HLA-A and HLA-B alleles were comparable between patients recruited during first or second wave of the COVID-19 epidemic (Data file S2).

### HLA class I and HLA class II binding prediction for T cell epitopes

Putative HLA restrictions were inferred using the Immune Epitope Database (IEDB, http://tools.iedb.org/main/). For HLA class I, all peptides included in the WT and Beta pools (Table S1) were submitted to Tepitool (http://tools.iedb.org/tepitool/) using the NetMHCpan method, including all HLA-A and HLA-B alleles identified in the study cohort (Data file S2). The epitopes that had a predicted IC50 > 50 nM were excluded and sequences ordered by percentile rank (67). For class II, the same peptides were submitted to the IEDB MHC class II epitope prediction tool (http://tools.iedb.org/mhcii/) including all HLA-DP, DQ and DR alleles identified in the cohort (Data file S1). HLA class II binding predictions were performed using two methodologies: first, NetMHCIIpan 3.2 (68, 69) was used to extract IC50 predicted values, then the IEDB recommended 2.22 methodology (combining the comblib (70), SMM (71), NN (68) and Sturniolo (72) algorithms was used and rank percentile values were extracted. Only epitopes with a predicted IC50 < 500 nM and percentile rank ≤ 20 were selected.

### Statistical analyses

Analyses were performed in Prism (v9; GraphPad). Nonparametric tests were used for all comparisons. The Mann-Whitney and Wilcoxon tests were used for unmatched and paired samples, respectively. *P* values less than 0.05 were considered to indicate statistical significance. All data used to compile figures can be found in Data file S4.

## Supplementary Material

Data file S4

Data file S3

Data file S2

Data file S1

Supplementary Figures

## Data Availability

All data are available in the main text or the supplementary materials. All materials used in this manuscript are commercially available.
