# Supplementary material for: Escape from recognition of SARS-CoV-2 Beta variant spike epitopes but overall preservation of T cell immunity
Source: Sci Transl Med. Author manuscript; Available in PMC 2022 Sep 1. (PMC9434381; doi:10.1126/scitranslmed.abj6824)
Supplement: Supplementary Figures [file NIHMS1831042-supplement-Supplementary_Figures.pdf]

Science Translational Medicine
AAAS

Supplementary Materials for

Escape from recognition of SARS-CoV-2 variant spike epitopes but overall preservation of T cell immunity

Catherine Riou *et al.*

Corresponding author: Catherine Riou, c.r.riou@uct.ac.za; Wendy A. Burgers, wendy.burgers@uct.ac.za

Sci. Transl. Med. **14**, eabj6824 (2022)
DOI: 10.1126/scitranslmed.abj6824

The PDF file includes:

Figs. S1 to S5
Tables S1 to S5

Other Supplementary Material for this manuscript includes the following:

Data files S1 to S4
MDAR Reproducibility Checklist

Supplementary Figure S1

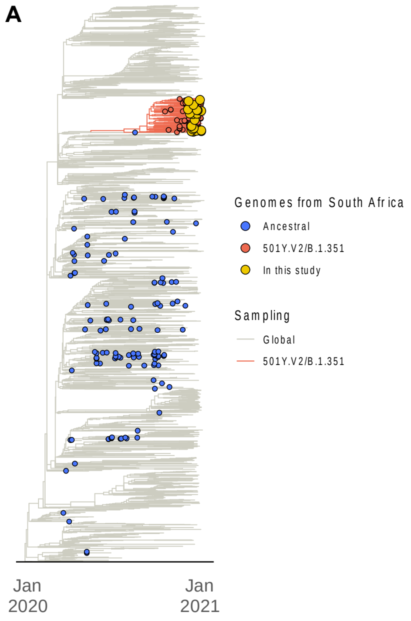

B

	Position & amino acid								
	18	80	215	242-244	246	417	484	501	701
**Ancestral**	L	D	D	LAL	R	K	E	N	A
**Beta**	F	A	G	- - -	I	N	K	Y	V
SA2-002	F	A	G	- - -	R	N	K	Y	V
SA2-006	L	A	G	- - -	R	N	K	Y	V
SA2-011	L	A	G	- - -	R	N	K	Y	V
SA2-012	L	A	G	- - -	R	N	K	Y	V
SA2-013	L	A	G	- - -	R	N	K	Y	V
SA2-017	L	A	G	- - -	R	N	K	Y	V
SA2-020**	L	A	G	- - -	R	N	K	Y	V
SA2-024	L	A	G	- - -	R	N	K	Y	V
SA2-029**	L	A	D	- - -	I	N	K	Y	V
SA2-037	L	A	G	- - -	R	N	K	Y	V
SA2-042	F	A	G	- - -	R	N	K	Y	V
SA2-043	F	A	G	- - -	R	N	K	Y	V
SA2-056	F	A	G	- - -	R	N	K	Y	V
SA2-070	F	A	G	- - -	R	N	K	Y	V
SA2-084	F	A	G	- - -	R	N	K	Y	V
SA2-094*	L	A	G	- - -	R	N	K	Y	V
SA2-095	F	A	G	- - -	R	N	K	Y	V
SA2-100	F	A	G	- - -	R	N	K	Y	V
SA2-102	F	A	G	- - -	R	N	K	Y	V
SA2-003	No sequence available								
SA2-016	No sequence available								
SA2-052	No sequence available								

Fig. S1. Genomic sequencing confirmation of SARS-CoV-2 Beta infection of COVID-19 second wave patients. (A) A time-resolved maximum clade credibility phylogeny of 2621 global SARS-CoV-2 sequences. Sequences from South Africa (n=209) are denoted with tip points. The Beta cluster is highlighted in red, and the genomes from this study (n=19) are shown in yellow, all falling in that cluster. **(B)** Spike sequence in patients recruited during the second wave, indicating amino acid changes. Blue shading corresponds with the ancestral strain (i.e., wild type, WT) amino acids and red shading to Beta variant amino acids. (-) corresponds to amino acid deletions. **: Patients exhibiting a detectable CD4 T cell response to the WT and Beta peptide pools, *: Patient exhibiting a detectable CD4 T cell response to the WT peptide pool.

Supplementary Figure S2

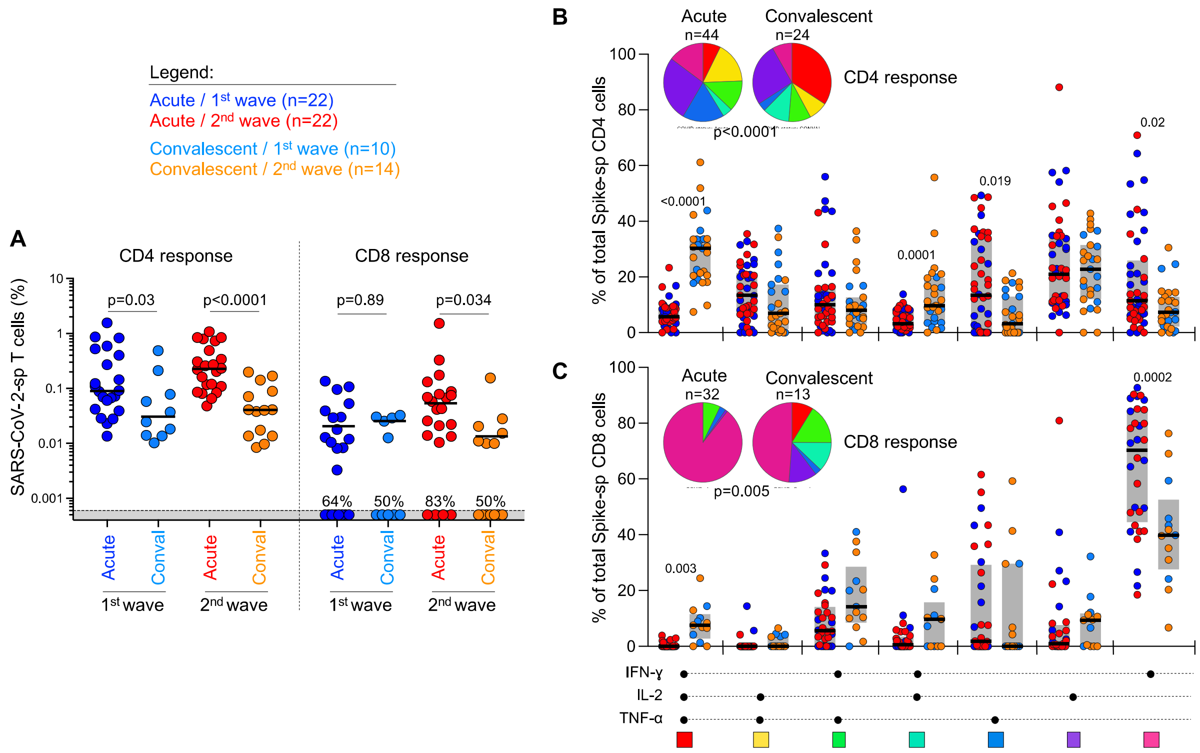

Fig. S2. Comparison of the frequency and polyfunctionality of T cell responses to ancestral SARS-CoV-2 Spike protein between acute and convalescent COVID-19 patients. (A) Summary graph of the frequency of ancestral SARS-CoV-2 Spike-specific CD4 and CD8 T cells, producing IFN-γ, TNF-α or IL-2, in acute and convalescent (~3 months post positive PCR) COVID-19 patients recruited during the first wave or second COVID-19 wave. The proportion of participant exhibiting a detectable CD8 response is indicated on the graph. Bars represent medians of responders. Statistical analyses were performed using the Mann-Whitney test including only participants with a detectable response. **(B&C)** Polyfunctional profile of ancestral Spike-specific CD4 (top) and CD8 (bottom) T cells in acute and convalescent COVID-19 patients. The medians and IQR are shown. Each response pattern is color-coded, and data are summarized in the pie charts. Statistical differences between pies were defined using a permutation test.

Supplementary Figure S3

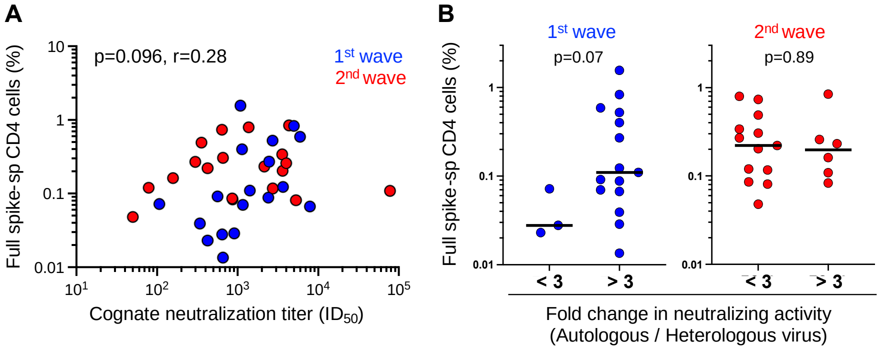

Fig. S3. Relationship between SARS-CoV-2-specific CD4 T cell responses and neutralizing activity. (A) Correlation between the frequency of ancestral SARS-CoV-2Spike-specific CD4 T cells and neutralization titers against cognate virus in 1st and 2nd wave patients. The correlation was tested by a two-tailed non-parametric Spearman rank test. **(B)** Comparison of the magnitude of ancestral SARS-CoV-2 Spike-specific CD4 T cell responses in patients exhibiting a fold change <3 or >3 in neutralizing activity against heterologous virus. Statistical analyses were performed using the Mann-Whitney test.

Supplementary Figure S4

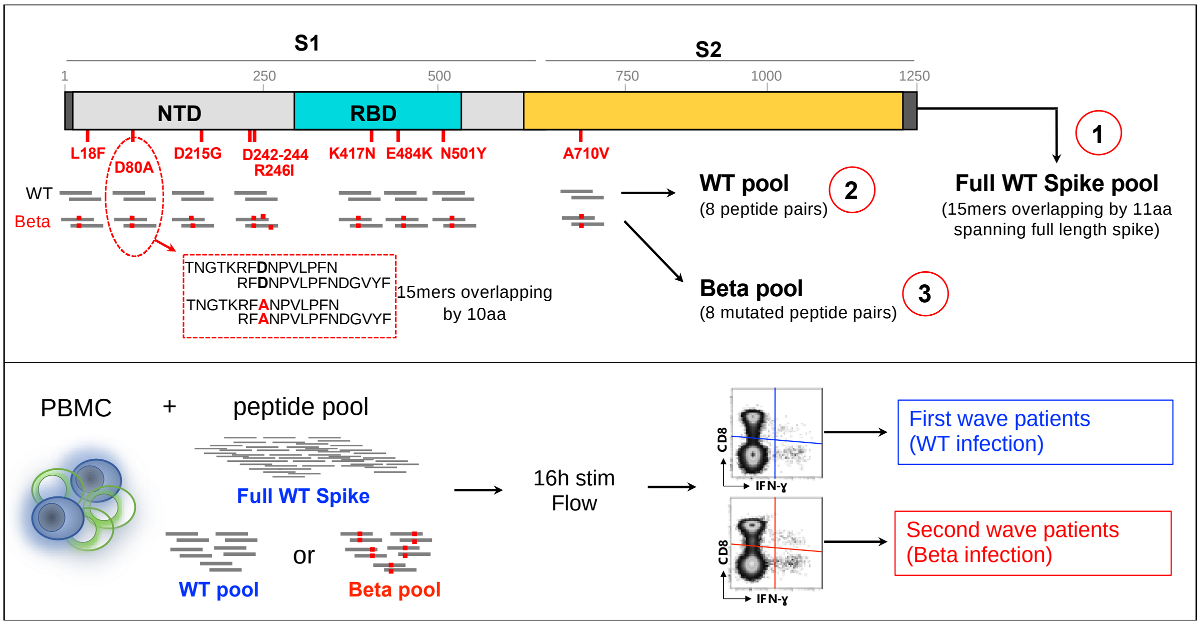

Fig. S4. Graphical representation of study approach. SARS-CoV-2 Spike protein is depicted, demonstrating the design of ancestral and Beta variant peptides used in the immunological assays. PBMC from patients recruited during the first or second wave of the pandemic in South Africa were stimulated with pools of peptides covering full Spike, or smaller pools spanning only the regions mutated in Beta, with wild type (WT) and corresponding mutated versions.

Supplementary Figure S5

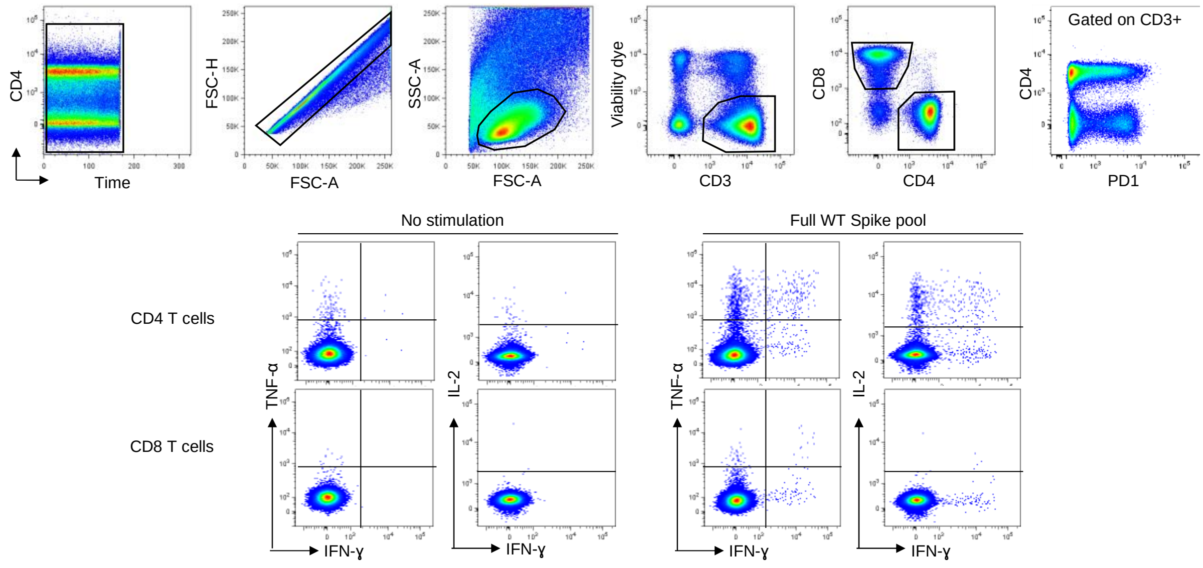

Fig. S5: Flow cytometry gating strategy. Representative example of flow cytometry staining profile and gating strategy.

Supplemental Table S1

Mutations of interest	Strain	Position aa (start)	Position aa (stop)	Sequence	Previously described
L18F	ancestral	6	20	VLLPLVSSQCVNLTT	Mateus et al.
L18F	ancestral	11	25	VSSQCVNLTTRTQLP	Tarke et al.
D80A	ancestral	73	87	TNGTKRFDNPVLPFN	
D80A	ancestral	78	92	RFDNPVLPFNDGVYF	Tarke et al.
D215G	ancestral	206	220	KHTPINLVRDLPQGF	Tarke et al.
D215G	ancestral	208	222	TPINLVRDLPQGFSA	Peng et al.
D215G	ancestral	211	225	NLVRDLPQGFSALEP	Peng et al.
242-244del/R246I	ancestral	236	250	TRFQTLLALHRSYLT	Mateus et al.
242-244del/R246I	ancestral	241	255	LLALHRSYLTPGDSS	Mateus et al.
K417N	ancestral	411	425	APGQTGKIADYNYKL	
K417N	ancestral	416	430	GKIADYNYKLPDDFT	Mateus et al.
E484K	ancestral	476	490	GSTPCNGVEGFNCYF	
E484K	ancestral	481	495	NGVEGFNCYFPLQSY	
N501Y	ancestral	492	506	LQSYGFQPTNGVGYQ	
N501Y	ancestral	496	510	GFQPTNGVGYQPYRV	
A701V	ancestral	701	715	AENSVAYSNNSIAIP	Mateus et al.
L18F	Beta	6	20	VLLPLVSSQCVNFTT	
L18F	Beta	11	25	VSSQCVNFTTRTQLP	
D80A	Beta	73	87	TNGTKRFANPVLPFN	
D80A	Beta	78	92	RFANPVLPFNDGVYF	
D215G	Beta	206	220	KHTPINLVRGLPQGF	
D215G	Beta	208	222	TPINLVRGLPQGFSA	
D215G	Beta	211	225	NLVRGLPQGFSALEP	
242-244del/R246I	Beta	236	250	TRFQTLHISYLTPGD	
242-244del/R246I	Beta	241	255	LHISYLTPGDSSSGW	
K417N	Beta	411	425	APGQTGNIADYNYKL	
K417N	Beta	416	430	GNIADYNYKLPDDFT	
E484K	Beta	476	490	GSTPCNGVKGFNCYF	
E484K	Beta	481	495	NGVKGFNCYFPLQSY	
N501Y	Beta	492	506	LQSYGFQPTYGVGYQ	
N501Y	Beta	496	510	GFQPTYGVGYQPYRV	
A701V	Beat	701	715	VENSVAYSNNSIAIP	

Table S1: List and sequence of 15-mer peptides contained in the ancestral (WT) and Beta peptide pools. References of previously described reactive peptides are provided in the last column. aa: amino acid. The site of mutations in Beta is underlined in the peptide sequence.

Supplemental Table S2

Mutations of interest	Strain	aa (start)	Sequence	Predicted HLA class II restriction
L18F	WT	6	VLLPLVSSQCVNLTT	DRB1*04:05, *15:01, *04:03
	Beta	6	VLLPLVSSQCVNFTT	DRB1*04:05, *15:01, *10:01
	WT	11	VSSQCVNLTTRTQLP	no match
	Beta	11	VSSQCVNFTTRTQLP	no match
D80A	WT	73	TNGTKRFDNPVLPFN	no match
	Beta	73	TNGTKRFANPVLPFN	no match
	WT	78	RFDNPVLPFNDGVYF	no match
	Beta	78	RFANPVLPFNDGVYF	no match
D215G	WT	206	KHTPINLVRDLPQGF	DRB1*03:01, *13:01, *13:02,
	Beta	206	KHTPINLVRGLPQGF	DRB1*11:04, *14:54, *04:03, *01:02, *11:01
	WT	208	TPINLVRDLPQGFSA	DRB1*03:01, *03:02, *13:01, *13:02, *14:54,
	Beta	208	TPINLVRGLPQGFSA	DRB1*11:04, *14:54, *08:04, *04:03, *01:02, *11:01
	WT	211	NLVRDLPQGFSALEP	DRB1*03:01, *13:02
	Beta	211	NLVRGLPQGFSALEP	DRB1*11:02
242-244del /R246I	WT	236	TRFQTLLALHRSYLT	DRB1*15:03, *12:02, *10:01, *14:24, *14:54, *01:01, *04:03, *15:01
	Beta	236	TRFQTLHISYLTPGD	DRB1*10:01, *01:01, *04:05, *07:01
	WT	241	LLALHRSYLTPGDSS	DRB1*15:01, *15:03, *07:01, *14:54, *14:25, *01:01
	Beta	241	LHISYLTPGDSSSGW	no match
K417N	WT	411	APGQTGKIADYNYKL	no match
	Beta	411	APGQTGNIADYNYKL	no match
	WT	416	GKIADYNYKLPDDFT	no match
	Beta	416	GNIADYNYKLPDDFT	no match
E484K	WT	476	GSTPCNGVEGFNCYF	no match
	Beta	476	GSTPCNGVKGFNCYF	no match
	WT	481	NGVEGFNCYFPLQSY	DQB1*05:02, DPB1*02:01
	Beta	481	NGVKGFNCYFPLQSY	DQB1*05:01, DPB1*02:01, DRB1*15:01,
N501Y	WT	492	LQSYGFQPTNGVGYQ	DRB1*09:01, *07:01, *04:01
	Beta	492	LQSYGFQPTYGVGYQ	DRB1*09:01, *07:01, *10:01
	WT	496	GFQPTNGVGYQPYRV	no match
	Beta	496	GFQPTYGVGYQPYRV	no match
A701V	WT	701	AENSVAYSNNSIAIP	DQB1*03:19, *03:01, DRB1*13:02, *04:01
	Beta	701	VENSVAYSNNSIAIP	DQB1*03:19, *03:01, DRB1*13:02, *04:01

Table S2: List of CD4 T cell epitopes used in this study and their predicted HLA class II restriction(s). Putative HLA class II restrictions were inferred using the Immune Epitope Database (IEDB) analysis resource (http://tools.iedb.org/mhcii/), using the IEDB recommended 2.22 prediction method. Prediction analyses were performed using all HLA class II (DR, DP, DQ) expressed in the studied cohort.

Supplemental Table S3

Wave	PID	HLA-DRB1		HLA-DQB1		HLA-DPB1	
1	SA1-001 #	DRB1*08:01	DRB1*13:01	DQB1*04:02	DQB1*06:03	DPB1*04:01	DPB1*04:02
1	SA1-002 #	DRB1*09:01	DRB1*15:01	DQB1*03:03	DQB1*06:03	DPB1*04:02	DPB1*14:01
1	SA1-005	DRB1*03:01	DRB1*07:01	DQB1*02:01	DQB1*02:02	DPB1*105:01	DPB1*105:01
1	SA1-007	DRB1*04:05	DRB1*13:02	DQB1*03:02	DQB1*06:09	DPB1*02:01	DPB1*17:01
1	SA1-008	DRB1*04:05	DRB1*12:02	DQB1*03:01	DQB1*03:02	DPB1*04:01	DPB1*13:01
1	SA1-015 #$	DRB1*13:01	DRB1*13:02	DQB1*06:03	DQB1*06:04	DPB1*01:01	DPB1*03:01
1	SA1-026	DRB1*04:04	DRB1*07:01	DQB1*03:02	DQB1*03:03	DPB1*04:01	DPB1*105:01
1	SA1-030	DRB1*11:01	DRB1*15:01	DQB1*03:01	DQB1*06:02	DPB1*04:01	DPB1*11:01
1	SA1-032 #	DRB1*13:03	DRB1*15:03	DQB1*06:02	DQB1*06:09	DPB1*02:02	DPB1*02:02
1	SA1-035 #$	DRB1*03:02	DRB1*12:01	DQB1*04:02	DQB1*05:01	DPB1*01:01	DPB1*131:01
1	SA1-043	DRB1*11:04	DRB1*11:14	DQB1*03:03	DQB1*03:03	DPB1*01:01	DPB1*105:01
1	SA1-049	DRB1*11:01	DRB1*11:01	DQB1*06:02	DQB1*06:02	DPB1*104:01	DPB1*104:01
1	SA1-066	DRB1*07:01	DRB1*08:04	DQB1*03:03	DQB1*04:02	DPB1*13:01	DPB1*105:01
1	SA1-068	DRB1*07:01	DRB1*11:01	DQB1*02:02	DQB1*06:02	DPB1*01:01	DPB1*02:01
1	SA1-075 #	DRB1*03:02	DRB1*15:01	DQB1*04:02	DQB1*06:01	DPB1*01:01	DPB1*02:01
1	SA1-087 #	DRB1*01:01	DRB1*04:03	DQB1*03:02	DQB1*05:01	DPB1*02:01	DPB1*04:02
1	SA1-090 #$	DRB1*12:02	DRB1*13:01	DQB1*03:01	DQB1*06:03	DPB1*02:01	DPB1*31:01
1	SA1-096 #$	DRB1*11:01	DRB1*14:54	DQB1*06:02	DQB1*06:02	DPB1*55:01	DPB1*104:01
1	SA1-098 #	DRB1*07:01	DRB1*14:25	DQB1*03:01	DQB1*03:03	DPB1*03:01	DPB1*04:01
1	SA1-099 #	DRB1*03:02	DRB1*11:02	DQB1*04:02	DQB1*06:09	DPB1*01:01	DPB1*01:01
1	SA1-155 #$	DRB1*10:01	DRB1*13:01	DQB1*05:01	DQB1*06:03	DPB1*02:01	DPB1*15:01
1	SA1-156	DRB1*04:03	DRB1*11:01	DQB1*03:01	DQB1*03:02	DPB1*04:01	DPB1*04:01
1	SA1c-004 #$	DRB1*03:01	DRB1*11:02	DQB1*02:01	DQB1*03:01	DPB1*02:01	DPB1*105:01
2	SA2-002	DRB1*01:02	DRB1*13:01	DQB1*05:01	DQB1*06:03	DPB1*03:01	DPB1*105:01
2	SA2-003	DRB1*08:04	DRB1*12:01	DQB1*03:01	DQB1*03:19	DPB1*02:01	DPB1*105:01
2	SA2-006	DRB1*07:01	DRB1*15:01	DQB1*03:03	DQB1*05:02	DPB1*03:01	DPB1*13:01
2	SA2-011	DRB1*15:01	DRB1*15:03	DQB1*06:01	DQB1*06:02	DPB1*01:01	DPB1*02:01
2	SA2-012	DRB1*12:01	DRB1*15:01	DQB1*05:01	DQB1*06:01	DPB1*02:01	DPB1*105:01
2	SA2-013	DRB1*07:01	DRB1*15:01	DQB1*02:01	DQB1*06:01	DPB1*04:01	DPB1*105:01
2	SA2-016	DRB1*07:01	DRB1*10:01	DQB1*02:02	DQB1*05:01	DPB1*04:01	DPB1*17:01
2	SA2-017	DRB1*03:01	DRB1*08:04	DQB1*02:01	DQB1*03:19	DPB1*105:01	DPB1*105:01
2	SA2-020 #	DRB1*13:01	DRB1*15:01	DQB1*06:02	DQB1*06:03	DPB1*04:01	DPB1*05:01
2	SA2-024	DRB1*07:01	DRB1*12:02	DQB1*02:02	DQB1*05:02	DPB1*04:01	DPB1*131:01
2	SA2-029 #	DRB1*07:01	DRB1*12:02	DQB1*03:01	DQB1*03:03	DPB1*05:01	DPB1*09:01
2	SA2-037	DRB1*15:03	DRB1*15:03	DQB1*06:02	DQB1*06:02	DPB1*01:01	DPB1*02:01
2	SA2-042	DRB1*11:01	DRB1*13:01	DQB1*03:19	DQB1*05:01	DPB1*01:01	DPB1*13:01
2	SA2-043	nd	nd	nd	nd	nd	nd
2	SA2-052	DRB1*03:02	DRB1*10:01	DQB1*04:02	DQB1*05:01	DPB1*01:01	DPB1*01:01
2	SA2-056	nd	nd	nd	nd	nd	nd
2	SA2-070	nd	nd	nd	nd	nd	nd
2	SA2-084	DRB1*03:01	DRB1*04:07	DQB1*02:01	DQB1*03:02	DPB1*02:01	DPB1*105:01
2	SA2-094 #	DRB1*10:01	DRB1*13:01	DQB1*05:01	DQB1*06:03	DPB1*03:01	DPB1*04:01
2	SA2-095	DRB1*10:01	DRB1*10:01	DQB1*04:02	DQB1*05:01	DPB1*01:01	DPB1*01:01
2	SA2-100	DRB1*11:01	DRB1*13:03	DQB1*03:01	DQB1*03:19	DPB1*01:01	DPB1*835:01
2	SA2-102	DRB1*13:01	DRB1*13:02	DQB1*06:09	DQB1*06:09	DPB1*18:01	DPB1*18:01

Table S3: HLA class II genotype (DRB1, DQB1 and DPB1) of acute COVID-19 patients.
[#]: Patients with a detectable CD4 T cell response to WT pool, [$]: Patients tested for identification of epitopes. nd: not done.

Supplemental Table S4

Wave	Donor ID	Epitope response (ICS)	Potential response (HLA prediction)	HLA-DRB1		HLA-DQB1		HLA-DPB1	
1	SA1-090	D215		DRB1*12:02	DRB1***13:01**	DQB1*03:01	DQB1*06:03	DPB1*02:01	DPB1*31:01
1	SA1-155	D215		DRB1*10:01	DRB1***13:01**	DQB1*05:01	DQB1*06:03	DPB1*02:01	DPB1*15:01
1	SA1c-004	D215		DRB1***03:01**	DRB1*11:02	DQB1*02:01	DQB1*03:01	DPB1*02:01	DPB1*105:01
1	SA1-015	D215/L18		DRB1***13:01**	DRB1*13:02	DQB1*06:03	DQB1*06:04	DPB1*01:01	DPB1*03:01
1	SA1-035	D215/L18		DRB1***03:02**	DRB1*12:01	DQB1*04:02	DQB1*05:01	DPB1*01:01	DPB1*131:01
1	SA1-096	D80		DRB1*11:01	DRB1*14:54	DQB1*06:02	DQB1*06:02	DPB1*55:01	DPB1*104:01
1	SA1-075	nd	D215/R246	DRB1***03:02**	DRB1***15:01**	DQB1*04:02	DQB1*06:01	DPB1*01:01	DPB1*02:01
1	SA1-001	nd	D215	DRB1*08:01	DRB1***13:01**	DQB1*04:02	DQB1*06:03	DPB1*04:01	DPB1*04:02
1	SA1-099	nd	D215	DRB1***03:02**	DRB1*11:02	DQB1*04:02	DQB1*06:09	DPB1*01:01	DPB1*01:01
1	SA1-002	nd	R246	DRB1*09:01	DRB1***15:01**	DQB1*03:03	DQB1*06:03	DPB1*04:02	DPB1*14:01
1	SA1-032	nd	R246	DRB1*13:03	DRB1***15:03**	DQB1*06:02	DQB1*06:09	DPB1*02:02	DPB1*02:02
1	SA1-087	nd	R246	DRB1***01:01**	DRB1*04:03	DQB1*03:02	DQB1*05:01	DPB1*02:01	DPB1*04:02
1	SA1-098	nd	R246	DRB1*07:01	DRB1***14:25**	DQB1*03:01	DQB1*03:03	DPB1*03:01	DPB1*04:01
2	SA2-020	nd	?	DRB1*13:01	DRB1*15:01	DQB1*06:02	DQB1*06:03	DPB1*04:01	DPB1*05:01
2	SA2-029	nd	?	DRB1*07:01	DRB1*12:02	DQB1*03:01	DQB1*03:03	DPB1*05:01	DPB1*09:01
2	SA2-094	nd	?	DRB1*10:01	DRB1*13:01	DQB1*05:01	DQB1*06:03	DPB1*03:01	DPB1*04:01

Table S4. List of Spike epitopes tested and their predicted HLA Class II restriction. Red corresponds to D215 restriction and green to R246 restriction.

Supplemental Table S5

Wave	Donor ID	HLA-A		HLA-B		HLA-C	
1	SA1-001	A*02:01	A*32:01	B*27:05	B*44:02	C*02:02	C*05:01
1	SA1-002	A*02:01	A*24:07	B*40:01	B*52:01	C*03:04	C*12:02
1	SA1-005	A*01:01	A*03:01	B*15:10	B*81:01	C*03:04	C*18:01
1	SA1-007	A*29:01	A*30:02	B*18:01	B*58:01	C*07:04	C*07:18
1	SA1-008	A*24:07	A*74:01	B*15:03	B*27:06	C*02:10	C*03:04
1	SA1-015	A*02:01	A*34:02	B*07:02	B*44:03	C*04:01	C*07:02
1	SA1-026	A*11:01	A*32:106	B*44:03	B*56:01	C*01:02	C*02:10
1	SA1-030	A*11:01	A*32:01	B*07:02	B*35:03	C*04:01	C*07:02
1	SA1-032	A*29:01	A*30:02	B*07:02	B*18:01	C*07:02	C*07:04
1	SA1-035	A*29:02	A*68:02	B*15:10	B*42:01	C*03:04	C*17:01
1	SA1-043	A*02:01	A*03:01	B*15:01	B*35:02	C*04:01	C*04:01
1	SA1-049	A*03:01	A*68:02	B*15:10	B*15:10	C*08:04	C*08:04
1	SA1-066	A*01:01	A*11:01	B*27:06	B*57:01	C*03:04	C*06:02
1	SA1-068	A*24:02	A*30:02	B*18:01	B*44:03	C*07:04	C*07:06
1	SA1-075	A*02:01	A*68:01	B*40:06	B*42:01	C*15:02	C*17:01
1	SA1-087	A*24:02	A*24:07	B*07:02	B*52:01	C*07:02	C*12:02
1	SA1-090	A*24:02	A*34:01	B*18:01	B*40:06	C*07:04	C*12:02
1	SA1-096	A*43:01	A*68:02	B*15:10	B*58:02	C*06:02	C*08:04
1	SA1-098 **#**	A*24:02	A*33:03	**B*35:05**	B*52:01	C*04:01	C*12:02
1	SA1-099	A*03:01	A*68:02	B*18:01	B*52:01	C*05:01	C*16:01
1	SA1-155	A*02:01	A*29:01	B*07:05	B*44:02	C*05:01	C*15:05
1	SA1-156	A*03:01	A*11:01	B*35:03	B*52:01	C*04:10	C*12:03
1	SA1c-004	A*01:01	A*68:01	B*08:01	B*58:02	C*06:02	C*07:01
2	SA2-002	A*02:01	A*30:01	B*42:02	B*57:03	C*07:01	C*17:01
2	SA2-003	A*02:131	A*30:01	B*07:06	B*15:10	C*03:04	C*07:02
2	SA2-006	A*11:01	A*32:01	B*07:06	B*57:01	C*06:02	C*07:02
2	SA2-011	A*02:03	A*68:02	B*14:02	B*38:02	C*07:02	C*08:02
2	SA2-012	A*02:11	A*36:01	B*13:02	B*40:06	C*06:02	C*15:02
2	SA2-013	A*26:12	A*33:03	B*40:06	B*58:02	C*06:02	C*15:02
2	SA2-016 **#**	A*02:05	A*02:05	B*50:01	**B*53:01**	C*06:02	C*06:02
2	SA2-017	A*02:01	A*02:02	B*18:01	B*53:01	C*04:01	C*05:01
2	SA2-020	A*01:01	A*26:01	B*38:01	B*41:02	C*12:03	C*17:03
2	SA2-024	A*03:01	A*11:01	B*35:30	B*58:02	C*04:01	C*06:02
2	SA2-029	A*02:01	A*31:01	B*15:08	B*15:03	C*01:02	C*08:01
2	SA2-037	A*24:02	A*68:02	B*07:02	B*13:02	C*06:02	C*07:02
2	SA2-042	A*02:01	A*03:01	B*14:02	B*15:03	C*02:10	C*08:02
2	SA2-043	nd	nd	nd	nd	nd	nd
2	SA2-052	A*34:02	A*68:02	B*15:10	B*44:03	C*03:04	C*04:01
2	SA2-056	nd	nd	nd	nd	nd	nd
2	SA2-070	nd	nd	nd	nd	nd	nd
2	SA2-084 **#**	A*23:01	A*24:07	**B*35:05**	B*58:01	C*04:01	C*07:18
2	SA2-094	A*11:01	A*11:01	B*44:03	B*52:01	C*04:01	C*12:02
2	SA2-095	A*02:81	A*29:02	B*15:10	B*42:01	C*03:04	C*17:01
2	SA2-100	A*01:01	A*03:01	B*15:16	B*81:01	C*14:02	C*18:01
2	SA2-102	A*02:01	A*30:02	B*39:10	B*44:03	C*07:06	C*12:03

Table S5: HLA class I genotype (HLA-A, -B and -C) of COVID-19 patients.
#: Patients with a detectable CD8 T cell response to the WT and Beta pools.
